# Effect of the Winter Wheat Cheyenne 5A Substituted Chromosome on Dynamics of Abscisic Acid and Cytokinins in Freezing-Sensitive Chinese Spring Genetic Background

**DOI:** 10.3389/fpls.2017.02033

**Published:** 2017-11-29

**Authors:** Balázs Kalapos, Aliz Novák, Petre Dobrev, Pavel Vítámvás, Ferenc Marincs, Gábor Galiba, Radomira Vanková

**Affiliations:** ^1^Agricultural Institute, Centre for Agricultural Research, Hungarian Academy of Sciences, Martonvásár, Hungary; ^2^Festetics Doctoral School, Georgikon Faculty, University of Pannonia, Keszthely, Hungary; ^3^Institute of Experimental Botany, Academy of Sciences of the Czech Republic, Prague, Czechia; ^4^Department of Genetics and Plant Breeding, Crop Research Institute, Prague, Czechia; ^5^Agricultural Biotechnology Institute, National Agricultural Research and Innovation Centre, Gödöllő, Hungary

**Keywords:** cold treatment, freezing tolerance, abscisic acid, cytokinin, phytohormones, gene expression, *Triticum aestivum*

## Abstract

The effect of short- and long-term cold treatment on the abscisic acid (ABA) and cytokinin (CK) metabolism, and their main biosynthesis- and signaling-related genes were investigated in freezing-sensitive and freezing-tolerant wheat genotypes. Varieties Cheyenne and Chinese Spring substituted with the 5A Cheyenne chromosome, which represented freezing-tolerant genotypes, were compared with the freezing-sensitive Chinese Spring. Hormone levels and gene expression data indicated that the short- and long-term cold treatments are associated with specific regulation of the accumulation of cold-protective proteins and phytohormone levels, as well as the expression profiles of the hormone-related genes. The significant differences were observed between the genotypes, and between their leaf and crown tissues, too. The level of dehydrins, including WCS120 protein, and expression of *WCS120* gene were considerably higher in the freezing-tolerant genotypes after 21 days of cold treatment. Expression of *Cor14b* and *CBF14*, cold-responsive regulator genes, was increased by cold treatment in all genotypes, to higher extent in freezing-tolerant genotypes. Cluster analysis revealed that the tolerant genotypes had a similar response to cold treatment, regarding expression of the ABA and CK metabolic genes, as well as hormone levels in leaves. As far as hormone levels in crowns are concerned, however, the strongly freezing-tolerant Cheyenne variety clustered separately from the Chinese Spring and the substitution line, which were more similar to each other after both 1 and 21 days of cold treatment than to Cheyenne. Based on these results we concluded that the 5A chromosome of wheat might have both a direct and an indirect impact on the phytohormone-dependent cold-induced freezing tolerance. Based on the gene expression data, novel genetic markers could be developed, which may be used to determine the freezing tolerance level in a wide range of wheat varieties.

## Introduction

One of the major cereals at present is the hexaploid (AABBDD genomes) *Triticum aestivum* or common wheat (Dubcovsky and Dvorak, 2007), which combines the D genome from *Aegilops tauschii* with the AB genomes from tetraploid wheat and exhibits a broader adaptability to different environmental conditions in comparison with its predecessors. As *T. aestivum* is allohexaploid, containing A, B, and D genomes, all of its homeologous genes are triplicated. Consequently, common wheat tolerates the absence of one chromosome from any pair, without losing its fertility. This unique feature made it possible to develop a series of nullisomic lines from a moderate freezing-tolerant spring variety Chinese Spring (CS) (Sears, 1953). Using this nullisomic series, intervarietal chromosome substitution lines were created. In each of the substitution lines, one chromosome pair from the CS was replaced by the corresponding pair of chromosomes from the frost-resistant winter wheat variety Cheyenne (Ch). Freezing test under natural and artificial conditions (Sutka, 1981; Sutka et al., 1986) confirmed earlier observations, which indicated that the chromosomes of 5th homologous group of Ch carry major genes controlling frost resistance (Cahalan and Law, 1979; Roberts, 1986). The relationship of cold hardening period and the level of frost resistance in different CS/Ch chromosome substitution lines was also reported (Veisz and Sutka, 1989; Vágújfalvi et al., 1999). The biggest alteration between CS and CS(Ch5A) survival rate was registered after 19-day cold treatment, i.e., 19% in case of CS, 64% in case of CS(Ch5A), while Ch had 100% survival (Vágújfalvi et al., 1999). Replacing chromosome 5A of CS with the corresponding chromosome from Ch [CS(Ch5A)] increased the freezing tolerance of CS (Chen et al., 2009; Eagles et al., 2011).

A cluster of at least 12 C-repeat binding factor (CBF) genes, also known as dehydration responsive elements (DRE-binding factors), are in the *frost resistance 2* locus (*Fr-A2*) on the long arm of chromosome 5A (Stockinger et al., 1997; Liu et al., 1998; Miller et al., 2006; Campoli et al., 2009), and therefore they were postulated to be responsible for freezing tolerance in wheat. The increased freezing tolerance of CS(Ch5A) was associated with increased transcription of the Ch CBF genes in the CS genetic background (Vágújfalvi et al., 2005). The target genes of CBFs include different transcription factors and effector genes belonging to the Cold-regulated (Cor) gene superfamily, the activation of which increases freezing tolerance (Pecchioni et al., 2014; Park et al., 2015). Among the genes of the CBF-cluster localized in the *Fr-2* locus, *CBF14* has a decisive influence on freezing tolerance, both in wheat and barley (Vágújfalvi et al., 2005; Fricano et al., 2009; Soltész et al., 2013; Dhillon and Stockinger, 2013). It is well established, that CBFs induce the expression of *HvCOR14b* (Crosatti et al., 1996), and *TaWCS120* (Vazquez-Tello et al., 1998), moreover they bind to the promoters of several other cold-regulated or drought-inducible genes in *Triticeae* (Cattivelli et al., 2002). These genes are differentially expressed in freezing-sensitive and freezing-tolerant plants during cold acclimation (Sarhan et al., 1997; Crosatti et al., 2003; Vágújfalvi et al., 2003; Kosová et al., 2012).

The *Cor14b* gene encodes a polypeptide that is accumulated in the stroma fraction of the chloroplasts in the presence of light and its transcription was specifically induced by low temperature and light (Crosatti et al., 1999). The *Cor14b* was used as a marker gene to prove the effectiveness of cold acclimation in cereals (Knox et al., 2008; Dhillon et al., 2010; Gulyás et al., 2014). *WCS120* belongs to the dehydrin (LEA II) gene family. The encoded WCS120 protein is supposed to be involved in low temperature-induced physiological dehydration. The level of WCS120 was considered as a bio-marker of freezing tolerance in both cold-acclimated (Vítámvás et al., 2007) and non-acclimated (Holková et al., 2009; Vítámvás et al., 2010; Kosová et al., 2013) winter wheats.

The first reaction of wheat plants to the sudden drop of temperature is the arrest of growth, followed by the adjustment of metabolism to the new, cold environment (acclimation phase). Phytohormones play a pivotal role in the coordination of this acclimation process (Galiba et al., 2013). One of the key players initiating the hormonal changes is the CBF-regulon itself, e.g., by enhancing the accumulation of the growth-repressing DELLA proteins (Achard et al., 2008).

In this paper we compare hormone and gene expression responses to 1 day cold-shock from previous work of our group (Kalapos et al., 2016) and the data obtained after 21 days of cold (this work) as a function of the chromosome 5A of Cheyenne in the CS genetic background. There are several reasons to choose these particular sampling points. First of all, when we compared the phytohormone responses of two common wheat cultivars (Kosová et al., 2012) and more recently of two einkorn lines (Vanková et al., 2014) during cold acclimation, characteristic changes specific for the individual stress response phases were found, which allowed optimizing the sampling times. The biggest alterations in the transcriptomes of CS and CS(Ch5A) substitution line were detected after 1 day cold treatment (Kocsy et al., 2010; Kalapos et al., 2016). As described above, the Ch5A related cold hardiness is almost fully manifested after 21-day cold treatment, with the lowest CS background effect. During this cold acclimation period, the plants still stay in the vegetative phase, which allowed us to avoid the hormonal changes associated with the developmental phase transition.

The environmental signals initiating the cold acclimation process are perceived by the leaves, but the survival of the plants depends on the fitness of meristems in the crowns (Hoffman et al., 2010). Due to the different functions of these organs, it is possible to expect different responses during cold acclimation. Indeed, when we recently analyzed the metabolic profiles, redox changes and gene expression levels in the crowns and leaves of CS and CS(Ch5A) substitution line, substantial differences were observed (Juhász et al., 2015; Boldizsár et al., 2016). These findings prompted us to study the phytohormone changes in both organs. Taking into account the intensive cross-talk among different phytohormones during cold acclimation, we focused on the interaction between the key hormone in abiotic stress responses – abscisic acid (ABA) and cytokinins (CKs), the hormones playing decisive role in plant stress acclimation. The evaluation of their metabolite contents has been complemented by determination of the expression pattern of selected hormone biosynthesis- and signaling-related genes.

## Materials and Methods

### Plant Material and Treatments

Seeds of Chinese Spring, Cheyenne and CS(Ch5A) substitution line (Veisz and Sutka, 1989; Vágújfalvi et al., 1999) were germinated for 5 days and then grown for 2 weeks under conditions described in Kalapos et al. (2016). The seedlings were grown in half-strength modified Hoagland solution under 16 h illumination at 270 μmol m^-2^ s^-1^, 20/15°C day/night temperature and 70–75% relative humidity in a growth chamber (Conviron). Plants were then transferred to 4°C for a cold treatment, with no change in other growing parameters. Samples were taken from leaves and crowns before the beginning of the cold treatment (control), in the end of the 1st day (cold-shock) and after 21 days of cold (acclimation). Samples collected from three biological replicates were used for phytohormone determination, protein analysis and quantitation of gene expression.

### Determination of Gene Expression by Quantitative RT-PCR

Total RNA was isolated using the Direct-zol^TM^ RNA Miniprep Kit (Zymo Research) according to the manufacturer’s instructions. RNA was reverse transcribed by M-MLV reverse transcriptase and Oligo(dT)_18_ primer (both from Thermo Scientific) according to the protocol of the manufacturer. Real-time quantitative PCR was performed using a CFX96 Touch^TM^ Real-Time PCR Detection System (Bio-Rad) and gene-specific primers (Supplementary Table 1). Data analysis was performed as described by Kalapos et al. (2016). The relative quantities of the individual transcripts were calculated by the ΔΔ*C*t method using a phosphogluconate-dehydrogenase gene (UniGene ID: Ta30797) for normalization (Paolacci et al., 2009). The relative expression values (fold change) were converted to log2 values, clustered and visualized with the Gitools software (Perez-Llamas and Lopez-Bigas, 2011). Ranking of genes was defined with the following formula: relative change = ABS {AVERAGE [Cheyenne + Chinese Spring(Cheyenne 5A)] - Chinese Spring}.

### Hormone Analysis

Leaf and crown samples (ca 50 mg FW) were purified and analyzed according to Dobrev and Kamínek (2002) and Dobrev and Vankova (2012). Briefly, homogenized samples were extracted with cold methanol/water/formic acid (15/4/1 v/v/v). The following labeled internal standards (10 pmol/sample) were added: ^2^H_3_-PA (phaseic acid), ^2^H_3_-DPA (dihydrophaseic acid), ^2^H_4_-7OH-ABA, ^2^H_5_-ABA-GE (ABA-glucosyl ester) (NRC-PBI), ^2^H_6_-ABA, ^2^H_5_-transZ, ^2^H_5_-transZR, ^2^H_5_-transZ7G, ^2^H_5_-transZ9G, ^2^H_5_-transZOG, ^2^H_5_-transZROG, ^2^H_5_-transZRMP, ^2^H_3_-DHZ, ^2^H_3_-DHZR, ^2^H_3_-DZRMP,^2^ H_7_-DZOG, ^2^H_3_-DHZ9G, ^2^H_7_-DZOG, ^2^H_6_-iP, ^2^H_6_-iPR, ^2^H_6_-iP7G, ^2^H_6_- iP9G, ^2^H_6_-iPRMP (Olchemim). Extracts were purified using a mixed mode reverse phase-cation exchange SPE column (Oasis-MCX, Waters). ABA metabolites were eluted with methanol and CK metabolites with 0.35 M NH_4_OH in 60% methanol. Hormones were analyzed using HPLC (Ultimate 3000, Dionex) coupled to a hybrid triple quadrupole/linear ion trap mass spectrometer (3200 Q TRAP, Applied Biosystems). Three biological replicates were analyzed. The cold-treated/control ratios of measured phytohormones were converted to log2 values, clustered and visualized with the Gitools software (Perez-Llamas and Lopez-Bigas, 2011). Ranking of phytohormones was defined with the following formula: relative change = ABS {AVERAGE [Cheyenne + Chinese Spring(Cheyenne 5A)] - Chinese Spring}.

### Determination of WCS120 Protein Content

One gram (FW) of leaves or crowns were homogenized under liquid nitrogen with 4.5 mL extraction buffer (100 mM Tris-HCl, pH 8.8, containing “Complete EDTA-free Protease Inhibitor Cocktail Tablets,” Roche) using the mortar and pestle. The mixture was centrifuged twice at 20,000 *g* at 4°C for 20 min, then kept in boiling water for 15 min, cooled rapidly on ice and centrifuged at 20,000 *g* (4°C) for 20 min. Concentration of heat-stable proteins was determined according to Bradford (1976). The supernatants were then precipitated by cold acetone with 1% 2-mercaptoethanol (v/v) using 1:5 sample:acetone ratio (v/v). The pellet was then centrifuged at 20,000 *g* (4°C) for 20 min and dried. Dry samples were resolved in Laemmli Sample buffer prepared according to the Bio-Rad manual (Hercules). The sample equivalent to 0.4 mg of tissue FW per genotype were loaded on 10% sodium dodecyl sulfate polyacrylamide gel (SDS-PAGE) (Laemmli, 1970). For the estimation of the protein molecular weight, “Precision Plus Protein^TM^ Standards, All Blue” (Bio-Rad) were used.

**FIGURE 1:**
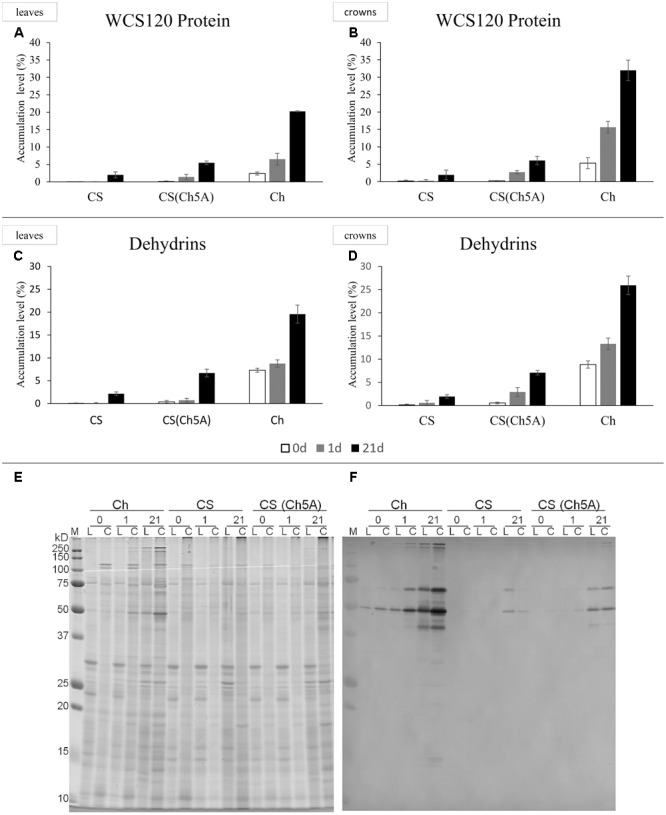
Accumulationx of WCS120 and dehydrins in leaves and crowns of cold-acclimated CS, CS(Ch5A) and Ch wheat genotypes. **(A,B)** Density of WCS120 was normalized by density of all loaded proteins in the same well (measured in Coomassie Blue stained gel). Sum of all WCS120 density values in the same biological replicate was recalculated as 100% accumulation. **(C,D)** Accumulation of dehydrins in wheat genotypes. Empty, gray and black columns show the results of untreated, 1- and 21-day cold treatments, respectively. **(E,F)** Gels stained with Coomassie are in the left, Western-blot is in the right. 0, control; 1, 1 day of cold; 21, 21 days of cold; C, proteins extracted from crowns; Ch, Cheyenne; CS, Chinese Spring, CS(Ch5A) – CS substitution line with 5A chromosome from Ch; L, proteins extracted from leaves; M, Precision Plus Protein^TM^ Standards, All Blue (Bio-Rad).

Protein gel blots were carried out on a semi-dry blotter (GE Healthcare) for 55 min at 0.8 mA cm^-2^ using a nitrocellulose membrane (pore diameter 0.45 μm; Bio-Rad). For hybridization with the antibody against dehydrin K-segment (Enzo Life Sciences, Inc.) to visualize dehydrins, an Immune-Blot Assay kit with Goat Anti-Rabbit IgG Alkaline Phosphatase (AP; Bio-Rad, Hercules) was used according to the manufacturer’s instructions. Membranes with visualized dehydrins were scanned by a calibrated densitometer GS-800 (Bio-Rad) at 600 dpi. Determination of the relative DHN protein accumulation was carried out using the Quantity One 4.6.7 program (Bio-Rad). Gels stained by Bio-Safe Coomassie (Bio-Rad, Hercules) were used for normalization of densities of dehydrin bends to quantity the loaded proteins (**Figure 1**).

## Results

### Expression of Genes Related to Cold Acclimation

**FIGURE 2:**
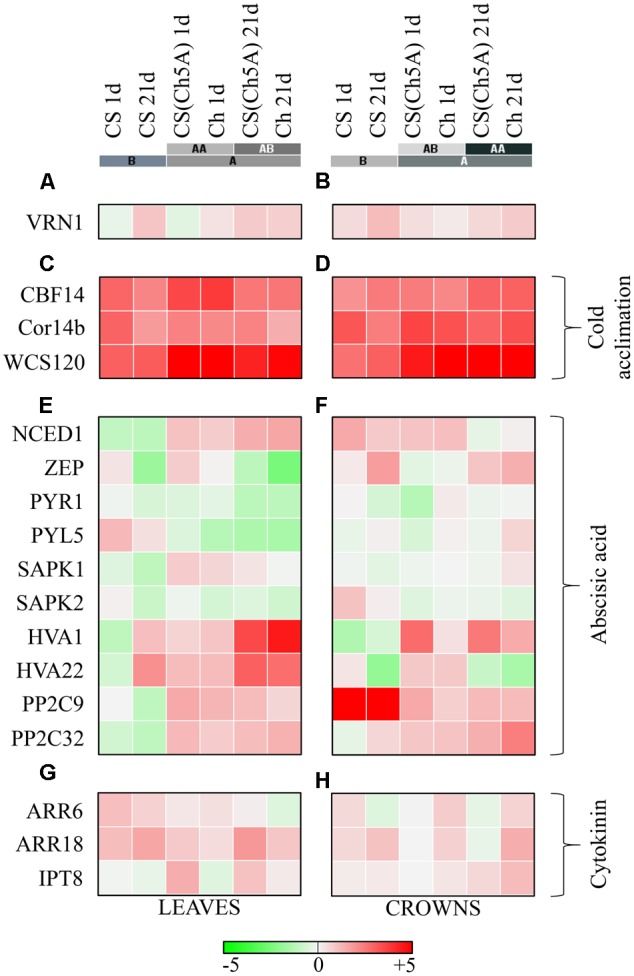
Heatmap visualization of the cold-induced gene expressions in the leaves and crowns of the three wheat genotypes. **(A,B)** *VRN1* Vernalization gene; **(C,D)** cold acclimation related genes; **(E,F)** abscisic acid metabolism and signal-transduction related genes; **(G,H)** cytokinin metabolism and signal-transduction related genes. Color scale represents the log2 transformed relative expression, which was calculated with the ΔΔ*C*t method. Colored bars with letters represent the hierarchical clusters in Euclidean distance. [*CBF14, NCED1, ZEP, PYR1, PYL5, SAPK1, SAPK2, PP29, PP2C32, ARR6, ARR18*, and *IPT8* gene expression data in 1 day cold treated leaves were presented in a previous study (Kalapos et al., 2016)].

In order to characterize the differences in cold stress responses among the studied genotypes and to prove the role of the chromosome 5A of Cheyenne in the improvement of freezing tolerance in the CS genetic background, expression profiles of four selected marker genes, *VRN1* (Vernalization 1) (**Figures 2A,B** and Supplementary Table 2), *CBF14* (C-repeat binding factor 14), *Cor14b* (Cold-responsive protein 14b) and *WCS120* (Wheat cold specific protein 120) were compared (**Figures 2C,D** and Supplementary Table 2).

After 1 day cold treatment, the expression of *VRN1* did not change significantly in the leaves of either genotype. After 21 days, approximately 5- and 3-fold increase in *VRN1* expression was detected in CS and CS(Ch5A) leaves, respectively, but only a low, non-significant elevation was detected in Cheyenne. Different expression patterns were observed in the crowns. In all examined genotypes, a slight increase of *VRN1* transcription was detected after 1 day, while about 2- to 4-fold *VRN1* elevation was found after 21 days (**Figures 2A,B** and Supplementary Table 2). The *VRN1* induction was stronger in CS than in the freezing-tolerant genotypes.

After 1 day cold treatment (Kalapos et al., 2016), expression of *CBF14* exhibited much higher increase in leaves of Ch and CS(Ch5A) genotypes than in CS (about 1000- and 500-fold, respectively, vs. about 70-fold). Although expression of *CBF14* decreased after 3 weeks of cold treatment in all genotypes, it remained still much higher in the freezing-tolerant genotypes than in CS (**Figures 2C,D** and Supplementary Table 2). In crowns, the expression of *CBF14* increased in all genotypes after 1 day cold-shock. The increase in the crowns was, however, considerably lower than in the leaves (Kalapos et al., 2016), with the lowest increase in CS. During acclimation, further increase of *CBF14* was observed, in CS comparable to that in leaves. The elevation in Ch and CS(Ch5A) was at least 4 times more profound than in CS.

After 1 day cold treatment, expression of the *Cor14b* highly increased in the leaves of all genotypes, especially in Ch. The transcript levels remained high only in the freezing-tolerant genotypes. The increase of transcript abundance was even higher in crowns, at least 600 times, 170 times and 100 times higher in Ch, CS(Ch5A) and CS, respectively, compared to the control. In the freezing-tolerant genotypes, the *Cor14b* transcript levels remained considerably higher than in the sensitive CS cultivar after 21 days (**Figures 2C,D** and Supplementary Table 2).

The amounts of *WCS120* transcripts were close to the detection limit in the control samples. After 1 day cold treatment, expression of *WCS120* increased almost 100-fold in the leaves of CS, while it was, in comparison with CS, at least 10 times higher in both Ch and CS(Ch5A) (**Figures 2C,D** and Supplementary Table 2). Although *WCS120* expression decreased after the 21-day treatment, the freezing-tolerant genotypes still contained 3- to 5-fold more transcripts than the sensitive CS. The elevation of transcript levels was also high in the crowns, but the pattern was different. The high values reached after 1 day cold-shock in the freezing-tolerant genotypes were further elevated after the long-term cold treatment. Crowns of Ch and CS(Ch5A) genotypes exhibited about 10- to 20-fold more *WCS120* transcripts than CS.

The level of WCS120 protein was generally in accordance with the gene expression pattern (**Figures 1A,B**). The freezing-tolerant genotypes accumulated higher levels of WCS120 than CS in all treatments. Under the control conditions, the level of WCS120 was clearly detectable in Ch, in contrast to the negligible WCS120 accumulation in the other two genotypes. After 1 day of cold treatment, significant increase (about 4-fold) of WCS120 was found only in Ch crowns. In crowns of all genotypes, the amount of WCS120 was higher than in their leaves at both time points, except in CS after 21-day treatment. After 3 weeks of cold treatment, the level of WCS120 in all genotypes correlated with their freezing tolerance, i.e., the highest and lowest WCS120 accumulation was found in freezing-tolerant Ch and freezing-sensitive CS, respectively, while in the moderately freezing-tolerant CS(Ch5A) the level of WCS120 was in-between.

### Expression of Genes Related to Abscisic Acid

In leaves, expression of *ZEP* (zeaxanthin epoxidase), which catalyzes an early step in ABA biosynthesis, increased after 1 day cold-shock (Kalapos et al., 2016), but decreased after 21 days of cold treatment in all genotypes (**Figure 2E** and Supplementary Table 2). The expression pattern correlated with the changes in ABA levels (see below). In contrast, the transcript level was changed in the opposite fashion in crowns (**Figure 2F** and Supplementary Table 2).

Expression of the *NCED1* (9-*cis*-epoxycarotenoid dioxygenase), coding the rate limiting ABA biosynthetic enzyme, highly correlated with determined ABA levels, both in leaves and crowns. *NCED1* expression was down-regulated in leaves of the freezing-sensitive CS variety during the whole cold treatment, while it was highly elevated in the freezing-tolerant genotypes (**Figure 2E** and Supplementary Table 2). In crowns, the transcript level was also up-regulated in all examined genotypes, but a slight reduction was detected in the freezing-tolerant genotypes after 21-day cold (**Figure 2F** and Supplementary Table 2).

Cold-shock down-regulated the expression of the ABA receptor gene *PYR1* (Pyrabactin resistance 1) in leaves (Kalapos et al., 2016). Further suppression of *PYR1* expression was observed after prolonged stress, especially in freezing-tolerant genotypes (**Figure 2E** and Supplementary Table 2). In crowns, such strong down-regulation was observed only in Ch (**Figure 2F** and Supplementary Table 2).

The expression of *PYL5* (PYR1-like protein 5) highly increased in the leaves of the freezing-sensitive variety CS (Kalapos et al., 2016), but decreased in the freezing-tolerant genotypes CS(Ch5A) and Ch (**Figure 2E** and Supplementary Table 2). In crowns, *PYL5* transcription was slightly down-regulated after 1 day cold-shock, increasing by the 21st day of the cold treatment in the leaves of all genotypes with the highest level measured in Ch variety (**Figure 2F** and Supplementary Table 2).

The SnRK2 kinase *SAPK1* (Stress-activated protein kinase 1), a component of the ABA signal transduction pathway was strongly down-regulated by short- and long-term cold treatment in leaves of the freezing-sensitive genotype CS (Kalapos et al., 2016), and was up-regulated in the freezing-tolerant genotypes (**Figure 2E** and Supplementary Table 2). In crowns, changes in *SAPK1* expression were not significant (**Figure 2F** and Supplementary Table 2).

Another serine/threonine-protein kinase gene, *SAPK2* (Stress-activated protein kinase 2) was down-regulated in leaves of all genotypes after 1 day cold treatment (Kalapos et al., 2016), declining further after 21 days (**Figure 2E** and Supplementary Table 2). Crowns exhibited a similar tendency, however, the transcription of *SAPK2* was stronger in CS compared to CS(Ch5A) and Ch (**Figure 2F** and Supplementary Table 2).

Expression of two ABA-inducible genes, *HVA1* (ABA-inducible protein HVA1) and *HVA22* (ABA-inducible protein HVA22), was down-regulated in CS leaves, while up-regulated in both tolerant genotypes after 1 day cold treatment. Both genes were up-regulated in all genotypes after 21 days. In crowns, the *HVA1* expression had the same pattern as in leaves. *HVA22* expression was up-regulated in Ch and CS, but not CS(Ch5A) line during the whole cold period. This suggests that the two genes might have diverse spatial and temporal regulation associated with their different roles in each genotype (**Figures 2E,F** and Supplementary Table 2).

The genes for two 2C-type protein phosphatases involved in ABA signaling, *PP2C9* (2C-type protein phosphatase protein C9) and *PP2C32* (2C-type protein phosphatase protein C32), were similarly down- and up-regulated in leaves of freezing-sensitive and tolerant genotypes, respectively, both after 1 (Kalapos et al., 2016) and 21 days of cold (**Figure 2F** and Supplementary Table 1). In crowns, however, *PP2C9* was strongly up-regulated in CS variety (at both time points). Expression of *PP2C32* was only slightly enhanced in CS. Moderate up-regulation of the expression of both phosphatase genes was observed in freezing-tolerant genotypes (**Figure 2E** and Supplementary Table 2).

### Expression of Genes Related to Cytokinins

The expression of a CK biosynthetic gene, *IPT8* (isopentenyl transferase 8), was up-regulated during the cold-shock in leaves of the substitution line (Kalapos et al., 2016) (**Figure 2G** and Supplementary Table 2). In crowns, moderate up-regulation of *IPT8* was observed in freezing-tolerant genotypes after the long-term cold (**Figure 2H** and Supplementary Table 2).

Expression of the negative, type-A response regulator *ARR6* was elevated by 1 day cold-shock in leaves (Kalapos et al., 2016), most strongly in CS. During acclimation, *ARR6* expression decreased, especially in Ch. Similar profile was observed in crowns, with exception of Ch, which maintained elevated *ARR6* expression even after 21 days.

Expression of the positive signaling component, type-B response regulator *ARR18* (transcription factor) was mildly increased in the leaves of all genotypes after 1 day cold (Kalapos et al., 2016) and strongly increased after 21-day stress (in Ch decreased). This tendency was detected also in crowns, with exception of CS(Ch5A), where no significant up-regulation was found.

### Dynamics of Abscisic Acid and Cytokinin Levels

In order to evaluate the impact of the substitution of the 5A chromosome of the spring wheat Chinese Spring by the 5A chromosome from the winter wheat Cheyenne, the hormone levels were measured after 1 day cold-shock (Kalapos et al., 2016) and 21-day acclimation period. The analyzed hormones included ABA and CK metabolites.

**FIGURE 3:**
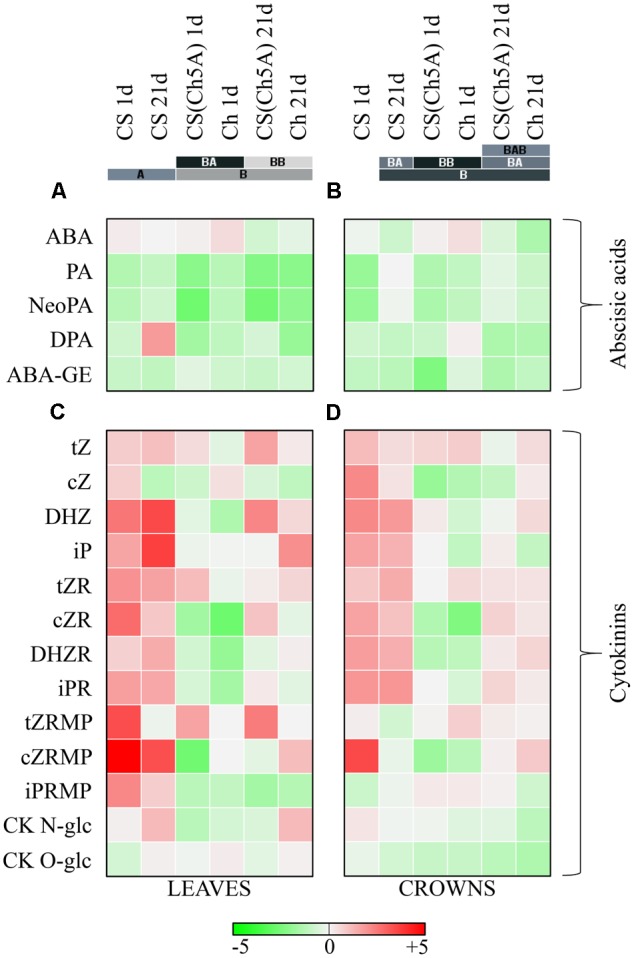
Heatmap visualization of the cold induced phytohormone levels in leaves and crowns of the three wheat genotypes. **(A,B)** Abscisic acid metabolites: ABA, abscisic acid; PA, phaseic acid; NeoPA, neophaseic acid; DPA, dihydrophaseic acid; ABA-GE, abscisic acid-glucosyl ester. **(C,D)** Cytokinin metabolites: tZ, *Trans*-zeatin; cZ, *cis*-zeatin; DHZ, Dihydrozeatin; iP, Isopentenyladenine; tZR; *Trans*-zeatin riboside; cZR, *cis*-zeatin riboside; DHZR, dihydrozeatin riboside; iPR, isopentenyladenosine; tZRMP, *Trans*-zeatin riboside monophosphate; cZRMP, *cis*-zeatin riboside monophosphate; iPRMP, isopentenyladenosine monophosphate; CK *N*-glc, cytokinin *N*-glucosides; CK *O*-glc, cytokinin *O*-glucosides. Color scale represents the log2 transformed cold treatment/control ratio of hormone levels. Colored bars with letters represent the hierarchical clusters in Euclidean distance. [Phytohormone accumulation data in 1 day cold treated leaves were presented in a previous study (Kalapos et al., 2016)].

The ABA increase was most profound in Ch leaves and crowns after 1 day cold (**Figures 3A,B** and Supplementary Table 3). The ABA elevation in CS(Ch5A) line was lower, while only moderate increase was found in CS leaves. Cold stress was generally associated with the decrease of ABA inactive metabolites phaseic acid (PA) and neophaseic acid (NeoPA) in leaves of all genotypes. PA increased only in CS and CS(Ch5A) crowns during prolonged stress. Dihydrophaseic acid (DPA) and ABA-glucosyl ester (ABA-GE) generally decreased in cold in all genotypes and tissues (**Figures 3A,B** and Supplementary Table 3).

The active CKs include *trans*-zeatin (tZ), isopentenyladenine (iP), dihydrozeatin (DHZ) and *cis*-zeatin (cZ). Cold-shock was associated with down-regulation of active CKs in leaves of the winter variety Ch (Kalapos et al., 2016), especially of tZ and DHZ. The level of cZ (CK with relatively low physiological activity, usually associated with growth-limiting conditions), however, was transiently increased. In contrast, the spring variety CS exhibited during cold-shock up-regulation of active CKs, including strong increase of cZ and DHZ. The CS(Ch5A) line exhibited intermediate response in leaves, cold-shock did not impose significant change in iP and only mild increase in tZ. As the level of cZ was down-regulated, the total amount of active CKs decreased. During acclimation, tZ and iP increased in leaves of all genotypes, which may indicate re-establishment of growth, however, cZ strongly decreased in comparison with control conditions (**Figure 3C** and Supplementary Table 3).

In crowns, mild decrease and no significant change of iP and tZ levels, respectively, were observed in Ch and CS(Ch5A) genotypes. The levels of cZ were strongly down-regulated. Only spring variety CS exhibited substantial elevation of total active CKs (especially during cold-shock) (**Figure 3D** and Supplementary Table 3).

Temperature decrease seems to be associated with the suppression of CK biosynthesis in Ch and CS(Ch5A) leaves, as indicated by decrease of precursors of active CKs – CK phosphates. In contrast, in CS leaves, highly significant up-regulation of CK phosphates was found, given mainly by elevation of cZRMP, which coincided with elevation of active CKs. After 21 days of cold, CK phosphates in the frost-tolerant genotypes [Ch and CS(Ch5A)] were still very low, while CS variety exhibited slightly higher level than the corresponding control plants (**Figure 3C** and Supplementary Table 3). In crowns, as meristematic tissues, CK phosphates were much more abundant than in leaves. No significant change in total CK phosphates was detected upon cold-shock in frost-tolerant genotypes. In case of CS, high increase of cZRMP was detected after 1 day cold (**Figure 3D** and Supplementary Table 3).

The CK ribosides represent transport forms, which are easily convertible into the active forms (bases). Frost-tolerant genotypes exhibited upon cold-shock decrease of CK ribosides in leaves, in contrast to CS, which substantially increased CK riboside levels, especially of cZR (Kalapos et al., 2016). After prolonged cold stress, Ch reached the control levels, the substitution line showed moderate elevation above the original value, while CS showed the highest increase. In crowns, cold-shock was associated with decrease of cZR only in frost-tolerant genotypes Ch and CS(Ch5A) (**Figures 3C,D** and Supplementary Table 3).

Down-regulation of CK biosynthesis in leaves of Ch and CS(Ch5A) genotypes was during cold-shock accompanied by decrease of CK deactivation by CK *N*-glucosylation (Kalapos et al., 2016). During acclimation, when active CKs were up-regulated, the levels of CK *N*-glucosides in leaves increased, probably in order to fine-regulate the CK pool. In crowns, gradual decrease of CK *N*-glucosides was found in Ch, mild up-regulation being found in CS. In contrast, CK *O*-glucosylation was upon cold-shock promoted predominantly in crowns of the spring variety CS, in leaves only after prolonged cold stress (**Figures 3C,D** and Supplementary Table 3).

## Discussion

The CBF regulon situated on the 5th chromosome of cereals has been known to play a pivotal role in the cold stress response in plants. We tested the impact of 5A chromosome on the cold reactions and freezing tolerance in wheat using the single chromosome substitution system. Since CBF genes, apart from the regulation of the Cor effector genes, also affect the hormonal homeostasis (Achard et al., 2008; Soltész et al., 2013), this substitution line provides a unique tool to elucidate the hormonal basis of effective cold acclimation. The effect of the 5A chromosome substitution on frost tolerance was reported to be associated with the function of the vernalization gene *VRN1*, which was mapped ∼30 cM apart from the *Fr-2* on the long arms of homologous group 5 chromosomes (Vágújfalvi et al., 2000, 2003, 2005). However, in our genetic system the expression profile of *VRN1* in CS and CS(Ch5A) was quite similar, both in leaves and crowns. So, the observed higher expression values of three freezing tolerance marker genes (*CBF14, Cor14b*, and *WCS120*) found in CS(Ch5A) lines were most likely due to the presence of Ch5A *Fr-2* locus in the CS genetic background and the potential modifying effect of *VRN1* was negligible. The expression values of the stress marker genes measured in CS(Ch5A) line were in between CS and Ch ones. The reason might be that apart from the 5A, also the 5D and to lesser extent 5B chromosomes are also effective in the performance of freezing tolerance, when originated from winter habit wheats (Sutka, 1981; Veisz and Sutka, 1989; Tóth et al., 2003; Pearce et al., 2013).

Comparing the tissue specific expression pattern of *CBF14* transcription factor, it is interesting to mention that although the expression level was considerably higher in leaves than in crowns after 1 day cold treatment, in the acclimation phase the transcript levels decreased in leaves, while further significantly increased in crowns. The expression level both in Ch and CS(Ch5A) was at every tested time point significantly higher than in CS. Thus, *CBF14* expression can be a good marker for freezing tolerance both in leaves and crowns during the cold stress response. In the case of the effector genes, the transcript level of *WCS120* seems to be much more useful positive marker of freezing tolerance than that of *Cor14b*. Independently from the treatment period, the *WCS120* transcript levels were significantly higher both in Ch and CS(Ch5A) genotypes than in CS. Although the expression level of *Cor14b* was significantly higher in Ch leaves and crowns in comparison with CS during the whole tested period, in case of CS(Ch5A) the *Cor14b* pattern was different. The *Cor14b* transcript level in the CS(Ch5A) crowns was higher after 1 day cold treatment, while in leaves it was higher after 21 days. In contrast to the transcript pattern of *WCS120*, which was very similar to that of *CBF14*, the *Cor14b* dynamics differed significantly. The reason might be that the *Cor14b* is regulated not only by *CBF* genes, but also by light (Crosatti et al., 1999).

### ABA Content and Expression of ABA Biosynthesis Genes

Temperature decrease is associated with the drop of the root hydraulic conductivity. The response to the resulting water deficit includes transient elevation of ABA. Short-term increase of ABA content was observed in leaves and crowns of all genotypes, most strongly in Ch. This transient ABA elevation is in accordance with previous studies on the hormonal responses to cold stress (Kosová et al., 2012; Vanková et al., 2014). Transient ABA up-regulation coincided with the elevation of the expression of gene coding for the rate limiting enzyme of ABA biosynthesis, *NCED1.* After cold-shock, *NCED1* expression was stimulated in crowns of all genotypes and in leaves of freezing-tolerant ones (Kalapos et al., 2016), where it was preserved during the whole stress treatment. In these genotypes, down-regulation of ABA levels after prolonged cold coincided in crowns with suppression of *NCED1* expression. The leaf samples of CS variety did not show difference between the short (Kalapos et al., 2016) and long-term cold treatments. Our data are in accordance with Wu et al. (2014), who found high increase of *NCED1* during cold acclimation (at 4°C) in grapevine. Stimulation of *NCED* expression was reported in case of several abiotic stresses, not only by cold. The up-regulation of *AtNCED* was found in drought-stressed Arabidopsis plants, where it was associated with the induced ABA levels (Xiong et al., 2002). *PaNCED1* from avocado was highly expressed in leaves after dehydration (Chernys and Zeevaart, 2000). Transient expression of *CrNCED1* in tobacco led to an increase in the ABA level as well as to enhanced tolerance to multiple abiotic stresses (Xian et al., 2014).

Expression of another ABA biosynthesis related gene, *ZEP*, displayed a different pattern in leaves and crowns. Transcription of *ZEP* was up-regulated in leaves upon cold-shock (Kalapos et al., 2016), while it was strongly enhanced in crowns after prolonged cold treatment (in all genotypes). This agrees with the report of Xiong et al. (2002), who found no up-regulation of *ZEP* expression in leaves of Arabidopsis plants after longer cold treatment. In *Medicago sativa* leaves too the expression of *MsZEP* considerably fell during the long-term cold treatment (Zhang H. et al., 2016). Our results revealed that the cold treatment-dependent down-regulation of *ZEP* in leaves and its up-regulation in crowns are associated with the organ-specific role in frost tolerance.

### ABA Signaling and Inducible Genes

The ABA signal transduction starts with the formation of a complex between ABA, PYR/PYL receptor protein and PP2C (repressor), which leads to the inhibition of PP2C activity and its degradation, resulting in the activation of SnRK2s. The activated SnRK2s then activate different downstream proteins, such as transcription factors and ion channels, which subsequently trigger ABA responses (Park et al., 2009; Gonzalez-Guzman et al., 2012). In our study, we followed expression of three core components of ABA signaling system – receptor genes (*PYR1, PYL5*), protein phosphatases 2C (*PP2C9, PP2C32*), the SnRK2 kinases (*SAPK1* and *SAPK2*) and two ABA-inducible genes coding LEA proteins (*HVA1, HVA22*). The expression of two ABA receptors, *PYR1* and *PYL5*, was strongly down-regulated in the leaves in cold and only slight enhancement was observed in the crowns of the freezing-tolerant genotypes. The expression of *SAPK2* was down-regulated in the freezing-sensitive leaves, and was up-regulated in the cold-shocked CS crowns. In parallel to this, the 2C-type protein phosphatases, *PP2C9* and *PP2C32*, were markedly up-regulated. These findings suggest that the suppression of ABA signaling, including the effect on ion channels and subsequently on stomata aperture, occurs in freezing-tolerant genotypes, especially after prolonged cold. However, it should be taken into consideration that the regulation of phosphatase and kinase activities is given by their phosphorylation/dephosphorylation, thus the expression pattern may not correlate precisely with their activities. From this point of view, relevant information may be obtained from the expression pattern of ABA-inducible genes. The up-regulation of genes coding LEA proteins, *HVA1* and *HVA22*, was delayed in CS leaves, being rapidly stimulated by cold-shock and further enhanced after prolonged stress in freezing-tolerant genotypes. In crowns, the expression was also higher in freezing-tolerant genotypes. These data suggest that ABA function in regulation of stomata aperture is important especially after the cold-shock. Later on, the effect of ABA on stimulation of the expression of protective proteins persists. This assumption is in accordance with the reports on positive effects of strengthened ABA signal transduction on the abiotic stress tolerance. Overexpression of PYR/PYL genes, such as *PYR1* and *PYL5*, enhanced the response to ABA in Arabidopsis plants and enhanced drought tolerance in rice (Santiago et al., 2009; Kim et al., 2014). TaSnRK2s were generally up-regulated by NaCl and cold treatment, with a maximal expression level at 6 h of the stress or later (Zhang Z. et al., 2016). *TaSnRK2C1* overexpression in transgenic tobacco increased the tolerance against dehydration, salt and low temperature (Du et al., 2013). Overexpression of other SnRK2s, such as *TaSNRK2.3, TaSnRK2.4, TaSnRK2.7*, and *TaSnRK2.8* resulted in enhanced tolerance against drought, salt, and low temperature (Mao et al., 2010; Zhang et al., 2010, 2011; Tian et al., 2013).

Special attention has been paid to ABA-inducible LEA (Late Embryogenesis Abundant) genes. These genes were detected in high abundance in the wheat dormant seeds (Sutton et al., 1992). *HVA1* mRNA was also highly accumulated after 6 h cold treatment in two freezing-resistant barley cultivars (Ried and Walker-Simmons, 1993). Overexpression of *HVA1* in transgenic rice significantly elevated the tolerance against water deficit and salt stress (Xu et al., 1996). In transgenic rice, *HVA1* was highly accumulated in root apical meristem and lateral root primordia, enhancing the tolerance to drought, salt and cold stresses (Chen et al., 2015). The barley *HVA1* overexpression in transgenic mulberry increased the drought, salt, and cold tolerance (Checker et al., 2012). Another LEA gene, *HVA22*, was directly induced by dehydration and cold stress both in shoots and roots of 3-day barley seedlings (Shen et al., 2001). *HVA22* was also rapidly induced by ABA and cycloheximide (Shen et al., 1993). Our results accord with the above mentioned studies and suggest that LEA genes, including *HVA1* and *HVA22*, play an important role during cold acclimation of wheat.

### Cytokinin Levels and Cytokinin- Related Genes

Cytokinins, phytohormones associated predominantly with stimulation of cell division and growth, have been recognized to play important roles in the stress responses, too (Ha et al., 2012).

Cytokinin’s growth promoting functions require dynamic and precise regulation of their levels and signal transduction during the cold stress progression. Application of cold-shock is accompanied with fast down-regulation of CK levels to allow growth suppression and re-allocation of energy sources from growth to the defense. Later on, during acclimation, CKs participate in growth re-initiation, exhibiting also positive effect on photosynthesis. In accordance with these hypotheses, active CKs (tZ, cZ, DHZ, and iP) in leaves and crowns of freezing-tolerant genotypes were decreased after cold-shock. This suppression was not observed in sensitive CS genotype (Kalapos et al., 2016). The expression of the negative, type-A response regulator, *ARR6*, was enhanced upon cold-shock in leaves (Kalapos et al., 2016) and crowns of all genotypes. This is in accordance with the reports of Vogel et al. (2005) and Jeon et al. (2010), who found a fast transient up-regulation of type-A response regulators, including *ARR6*, upon cold stress. Higher transcript level of *ARR6* in the leaves of cold-shocked freezing-sensitive CS genotype suggests that the function of *ARR6* is predominantly in regulation of CK signaling and rather indirectly in the cold stress response.

During acclimation, the levels of active CKs moderately increased. The expression of *ARR6* was down-regulated, in leaves predominantly in Ch, in crowns in CS and substitution line. Simultaneously, the expression of the positive, type-B response regulator, *ARR18*, was enhanced, to higher extent in leaves of CS and substitution line and in crowns in all genotypes (mostly in Ch). Elevation of active CK levels as well as their signal transduction indicates at least partial acclimation to the new environmental conditions, especially in freezing-tolerant genotypes. The importance of up-regulation of CK signaling pathway is indicated by the fact that loss-of-function *ARR6* mutants (i.e., mutants with promoted CK signal transduction) showed enhanced freezing tolerance in Arabidopsis (Jeon et al., 2010). CK profile during the cold stress progression is in accordance with reports of Kosová et al. (2012) and Vanková et al. (2014).

The key CK biosynthetic enzyme is isopentenyl transferase. In our experiments, we determined expression of *IPT8*. Surprisingly, it was enhanced in leaves of the substitution line and to a mild extent in crowns of all genotypes. The reason for the discrepancy between the profiles of active CKs and *IPT8* expression might be the presence of multiple genes for CK biosynthesis, each regulated in a specific way (there are 9 isogenes in Arabidopsis).

Our findings suggest that the expression of type-A *ARR6* and type-B *ARR18* response regulators seems not to be under the control of chromosome 5A. These two genes are similarly regulated in the CS(Ch5A) substitution line and in the genetic background genotype CS.

## Conclusion

**FIGURE 4:**
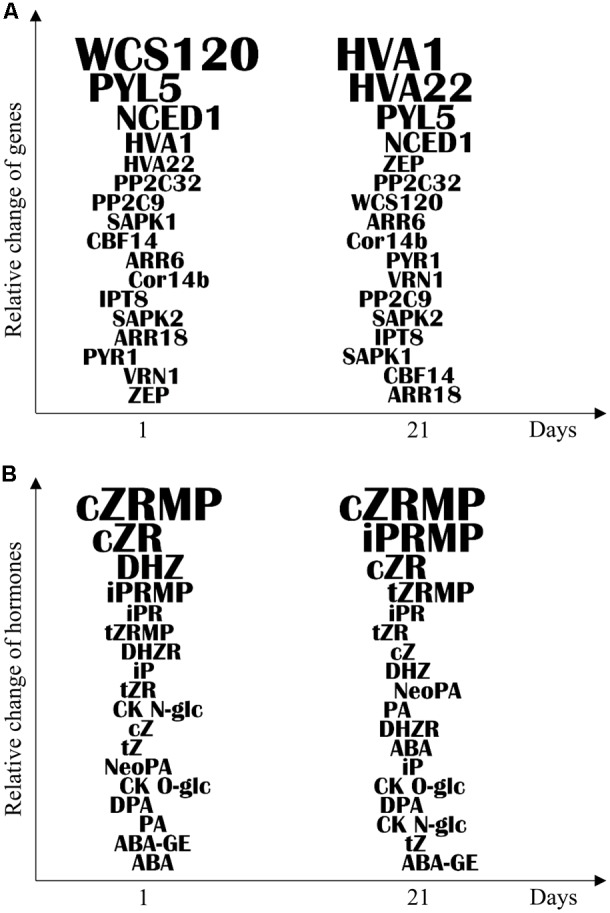
Ranking of differentially expressed genes and phytohormones according to the importance in the cold response in leaf samples. **(A)** Ranking of genes according to the expression level difference between freezing-tolerant [Ch; CS(Ch5A)] and freezing-sensitive (CS) genotypes following 1 and 21 days of cold treatment. **(B)** Ranking of phytohormones according to the accumulation level difference between the freezing-tolerant [Ch; CS(Ch5A)] and freezing-sensitive (CS) genotypes following 1 and 21 days of cold treatment. The formula for calculating the relative change was described in section “Material and Methods.” The letter size of genes and phytohormones represent the relative change level.

Evaluation of the role of chromosome 5 in freezing tolerance and regulation of hormone stress responses was performed comparing freezing-sensitive and freezing-tolerant genotypes with CS(Ch5A) substitution line. Based on our results we conclude that wheat chromosome 5A regulates, both directly and indirectly, the establishment of freezing tolerance. Apart of the already known effect on major cold inducible genes, including *CBF14, Cor14b*, and *WCS120*, we found that chromosome 5A is associated also with regulation of hormone-related genes and metabolites underlying the freezing tolerance. Using a systems biological approach, dynamics of the expression of freezing tolerance marker genes was correlated with the changes in phytohormone contents, as well as with transcription pattern of the hormone related genes. The most relevant differences between the two tolerant genotypes and CS may become useful as molecular markers of freezing tolerance (**Figures 4A,B**).

## Author Contributions

BK performed the gene expression experiments, analyzed the data, and wrote the manuscript. AN participated in data analysis. PD and RV performed hormone measurements. PV performed protein analysis. FM participated in data analysis. GG and RV conceived the study and participated in writing of the manuscript.

## Conflict of Interest Statement

The authors declare that the research was conducted in the absence of any commercial or financial relationships that could be construed as a potential conflict of interest.
