# Supplementary material for: Effect of the Winter Wheat Cheyenne 5A Substituted Chromosome on Dynamics of Abscisic Acid and Cytokinins in Freezing-Sensitive Chinese Spring Genetic Background
Source: Front Plant Sci. 2017 Nov 29;8:2033. doi: 10.3389/fpls.2017.02033 (PMC5712565; doi:10.3389/fpls.2017.02033)
Supplement: Supplementary file 1 [file Table_1.pdf]

Supplementary Table 1. Primers used for qRT-PCR.

Gene	Forward primer	Reverse primer	Reference
VRN1	GAACAAGATCAACCGGCAGGTGAC	GGAGAAGATGATGAGGCCGACCTC	Boldizsár et al., 2016
CBF14	CCACCAAATATGGGAGGAAA	CTTTCACAATGAACGAGCA	Dhillon et al., 2010
Cor14b	GAGCGACTCCTGCTAACGAC	CTACCGCCTCCTGTACCTTG	Dhillon et al., 2010
WCS120	CGTTGGAGGAGAGAATGTATTATG	CTGCGTCTGTCTCTTGGATAAG	Campoli et al., 2009
NCED1	ACGTGCCGGACTGCTTCTGCT	GCCGGTGCGCGTGTTGAG	Gulyas et al., 2014
ZEP	CCTTGCTGATGCCCGCGAACACGAG	CTGAGAGCGGTACCAAGATGAAG	Kalapos et al., 2016
PYR1	GACGGCAACAGCGAGGAGGACAC	GGCGACGGACTTGAGCTTCTGGAG	Kalapos et al., 2016
PYL5	CCGGCGGGCAACACGAT	TCACGCGCCGAGGAAACAC	Kalapos et al., 2016
SAPK1	GATCATCCAGGAGGCGCAGAAACC	GAGCAATCACAACGGGCACACGAA	Kalapos et al., 2016
SAPK2	TGTCCACTCCACTCGCCACTG	CCCTATGTCCTTGATCACCTCGTA	Kalapos et al., 2016
HVA1	GGCCGGCAAGGACAAGACC	ACGGCCGAACACGACTAAAGGAG	Kalapos et al., 2016
HVA22	GGCGCCTCCTTCATCTACGACA	CAGTGCCATGCCCCTTCTCCT	Kalapos et al., 2016
PP2C9	AGGGGGTGAGCAAGGCGGAGTC	CGAGAAGCAATCATGGAGCACGAA	Kalapos et al., 2016
PP2C32	CCGACTGCGAGCTGCTCATCCT	GCGCTGCAGCTGGATGACGAG	Kalapos et al., 2016
ARR6	TTCTTCGCCGTTCCATACTTCTGC	TCGTCGGCGTCGGTCATCA	Kalapos et al., 2016
ARR18	ACTCGCCCACCTCCCATCTCA	GCTCCGGCGTCCACACCAT	Kalapos et al., 2016
IPT8	CGCGCCAATGACCTCTTCTTCT	CGCCCACGATCACCACCAG	Kalapos et al., 2016
TA30797 (ref. gene)	GCCGTGTCCATGCCAGTG	TTAGCCTGAACCACCTGTGC	Paolacci et al., 2009
