# Supplementary material for: Effect of the Winter Wheat Cheyenne 5A Substituted Chromosome on Dynamics of Abscisic Acid and Cytokinins in Freezing-Sensitive Chinese Spring Genetic Background
Source: Front Plant Sci. 2017 Nov 29;8:2033. doi: 10.3389/fpls.2017.02033 (PMC5712565; doi:10.3389/fpls.2017.02033)
Supplement: Supplementary file 2 [file Table_2.pdf]

Supplementary Table 2. Relative expression of cold-responsive and hormone-related genes (log2 FC).

	LEAVES						CROWNS					
	CS		CS(Ch5A)		Ch		CS		CS(Ch5A)		Ch	
	1 d	21 d	1 d	21 d	1 d	21 d	1 d	21 d	1 d	21 d	1 d	21 d
Vernalization (Developmental) gene												
**VRN1**	-0.42	1.88	-0.76	1.69	0.68	1.47	0.98	2.25	0.92	1.10	0.43	1.68
Cold acclimation genes												
**CBF14**	5.84	4.51	7.06	5.12	7.62	5.14	3.98	4.99	4.90	5.91	4.44	5.95
**Cor14b**	5.94	3.66	4.71	4.66	4.35	2.87	6.48	4.86	7.22	5.90	6.65	6.72
**WCS120**	6.04	6.23	10.39	8.60	11.13	9.81	5.34	6.04	8.97	9.99	10.19	10.33
ABA-related genes												
**NCED1**	-2.06	-2.28	2.06	2.90	1.62	3.15	3.03	1.71	1.99	-0.52	2.18	0.19
**ZEP**	0.67	-3.59	1.60	-2.24	0.12	-5.04	0.47	3.55	-0.71	1.88	-0.31	2.81
**PYR1**	-0.16	-1.15	-0.98	-2.29	-0.61	-2.22	0.07	-1.22	-2.51	-0.30	0.41	-0.10
**PYL5**	2.42	0.83	-0.94	-2.92	-2.49	-3.06	-0.47	0.19	-1.17	-0.29	0.18	1.28
**SAPK1**	-0.85	-2.18	1.65	0.64	1.22	-0.07	-0.15	-0.65	-0.23	-0.20	-0.04	0.73
**SAPK2**	0.18	-1.74	-0.23	-0.92	-1.26	-1.50	2.00	0.28	-0.86	-0.39	-0.28	-0.87
**HVA1**	-2.17	2.19	1.33	7.01	1.90	8.96	-2.74	-1.25	5.54	5.08	0.78	2.97
**HVA22**	-1.36	4.08	2.29	6.05	2.32	5.47	0.62	-3.90	1.76	-1.77	1.79	-3.03
**PP2C9**	0.01	-2.19	3.09	2.29	2.55	1.23	12.61	11.41	3.07	2.36	1.51	2.28
**PP2C32**	-1.41	-2.14	2.43	2.24	1.63	2.72	-0.52	1.20	1.86	2.79	1.97	4.76
Cytokinin-related genes												
**ARR6**	2.18	1.38	0.53	0.27	0.84	-0.92	1.07	-0.90	0.03	-0.56	1.62	1.32
**ARR18**	2.22	3.17	1.72	3.74	0.93	1.84	1.15	2.03	-0.02	-0.43	1.45	2.88
**IPT8**	-0.09	-0.49	2.89	2.07	-0.86	0.40	0.37	0.45	-0.03	1.13	0.63	2.26
