# Supplementary material for: Effect of the Winter Wheat Cheyenne 5A Substituted Chromosome on Dynamics of Abscisic Acid and Cytokinins in Freezing-Sensitive Chinese Spring Genetic Background
Source: Front Plant Sci. 2017 Nov 29;8:2033. doi: 10.3389/fpls.2017.02033 (PMC5712565; doi:10.3389/fpls.2017.02033)
Supplement: Supplementary file 3 [file Table_3.pdf]

Supplementary Table 3. Cold-treatment/control ratio (log2 value) of phytohormone levels.

	LEAVES						CROWNS					
	CS		CS(Ch5A)		Ch		CS		CS(Ch5A)		Ch	
	1 d	21 d	1 d	21 d	1 d	21 d	1 d	21 d	1 d	21 d	1 d	21 d
	Abscisic acids (ABAs)											
**Abscisic acid (ABA)**	0.17	-0.03	0.11	-0.72	0.49	-0.31	-0.13	-0.78	0.11	-0.54	0.46	-1.44
**Phaseic acid (PA)**	-1.34	-0.99	-2.16	-2.29	-1.23	-2.16	-1.88	-0.01	-1.37	-0.36	-1.03	-0.86
**Neophaseic acid (NeoPA)**	-1.22	-0.74	-2.71	-2.60	-1.14	-2.01	-1.89	-0.11	-1.48	-0.38	-1.04	-0.83
**Dihydrophaseic acid (DPA)**	-0.77	1.84	-1.65	-0.62	-1.07	-1.88	-0.77	-0.97	-0.85	-1.47	0.12	-1.38
**Abscisic acid-glucosyl ester (ABA-GE)**	-0.90	-1.04	-0.39	-0.88	-0.77	-0.67	-1.02	-1.17	-2.37	-1.42	-0.50	-1.03
	Cytokinins (CKs)											
**Trans-zeatin (tZ)**	0.80	1.07	0.50	-0.37	1.65	0.23	1.16	0.50	0.63	0.81	-0.20	0.49
**Cis-zeatin (cZ)**	0.76	-1.15	-0.81	0.42	-0.55	-1.06	2.19	0.36	-1.88	-1.32	-1.00	0.22
**Dihydrozeatin (DHZ)**	2.57	3.50	-0.36	-1.40	2.24	0.57	2.18	1.86	0.20	-0.69	-0.11	0.54
**Isopentenyladenine (iP)**	1.63	3.69	-0.16	-0.05	-0.05	2.06	1.65	1.33	0.01	-1.03	0.19	-0.99
**Trans-zeatin riboside (tZR)**	1.99	1.66	1.13	-0.20	0.19	0.62	0.90	1.46	-0.01	0.54	0.36	0.34
**Cis-zeatin riboside (cZR)**	2.78	0.91	-1.66	-2.73	0.98	-0.30	1.65	1.02	-1.36	-2.35	0.67	0.28
**Dihydrozeatin riboside (DHZR)**	0.71	1.47	-0.72	-1.94	-0.40	0.14	1.76	1.41	-1.22	-1.10	0.24	0.63
**Isopentenyladenosine (iPR)**	1.73	1.57	-0.59	-1.61	0.22	-0.38	1.92	1.94	-0.02	-0.60	0.64	0.20
**Trans-zeatin riboside monophosphate (tZRMP)**	3.41	-0.15	1.64	0.00	2.44	0.00	0.15	-0.69	0.06	0.75	0.18	0.08
**Cis-zeatin riboside monophosphate (cZRMP)**	8.16	3.37	-2.65	0.00	-0.32	1.11	3.50	-0.21	-1.77	-1.17	0.12	0.86
**Isopentenyladenosine monophosphate (iPRMP)**	2.22	0.79	-1.18	-1.00	-1.63	-1.28	-0.83	-0.15	0.23	0.23	0.08	-0.77
**Cytokinin N-glucosides (CK N-glc)**	0.11	1.17	-1.19	-0.64	-0.51	1.18	0.30	-0.08	-0.11	-0.41	-0.37	-1.12
**Cytokinin O-glucosides (CK O-glc)**	-0.65	0.10	-0.09	0.17	-0.35	0.08	-0.22	-0.68	-0.88	-0.89	-1.16	-1.39
